# Economic effects of interventions to reduce obesity in Israel

**DOI:** 10.1186/2045-4015-1-17

**Published:** 2012-04-18

**Authors:** Gary M Ginsberg, Elliot Rosenberg

**Affiliations:** 1 Medical Technology Assessment Sector, Ministry of Health, Ben Tabai 2, Jerusalem, Israel; 2 Healthy Israel 2020 Initiative, Ministry of Health, Pierre Koenig 33, Jerusalem, Israel

## Abstract

**Background:**

Obesity is a major risk factor for many diseases. The paper calculates the economic impact and the cost per Quality-Adjusted Life Year (QALY) resulting from the adoption of eight interventions comprising the clinical and part of the community components of the National Prevention and Health Promotion Program (NPHPP) of the Israeli Ministry of Health (MOH) which represents the obesity control implementation arm of the MOH Healthy Israel 2020 Initiative.

**Methods:**

Health care costs per person were calculated by body mass index (BMI) by applying Israeli cost data to aggregated results from international studies. These were applied to BMI changes from eight intervention programmes in order to calculate reductions in direct treatment costs. Indirect cost savings were also estimated as were additional costs due to increased longevity of program participants. Data on costs and QALYs gained from Israeli and International dietary interventions were combined to provide cost-utility estimates of an intervention program to reduce obesity in Israel over a range of recidivism rates.

**Results:**

On average, persons who were overweight (25 ≤ BMI < 30)had health care costs that were 12.2% above the average health care costs of persons with normal or sub-normal weight to height ratios (BMI < 25). This differential in costs rose to 31.4% and 73.0% for obese and severely obese persons, respectively.

For overweight (25 ≤ BMI < 30) and obese persons (30 ≤ BMI < 40), costs per person for the interventions (including the screening overhead) ranged from 35 NIS for a community intervention to 860 NIS, reflecting the intensity of the clinical setting intervention and the unit costs of the professionals carrying out the intervention [e.g., dietician]. Expected average BMI decreases ranged from 0.05 to 0.90. Higher intervention costs and larger BMI decreases characterized the two clinical lifestyle interventions for the severely obese (BMI ≥ 40).

A program directed at the entire Israeli population aged 20 and over, using a variety of eight different interventions would cost 2.07 billion NIS overall. In the baseline scenario (with an assumed recidivism rate of 50% per annum), approximately 620,000,000 NIS would be recouped in the form of decreased treatment costs and indirect costs, increased productivity and decreased absenteeism. After discounting the 89,000,000 NIS additional health costs attributable to these extra life years, it is estimated that the total net costs to society would be 1.55 billion NIS. This total net cost was relatively stable to increases in the program's recidivism rates, but highly sensitive to reductions in recidivism rates.

Under baseline assumptions, implementation of the cluster of interventions would save 32,671 discounted QALYs at a cost of only 47,559 NIS per QALY, less than half of the Israeli per capita GNP (104,000 NIS). Thus implementation of these components of the NPHPP should be considered very cost-effective.

**Conclusion:**

Despite the large costs of such a large national program to control obesity, cost-utility analysis strongly supports its introduction.

## Background

The increasing prevalence of obesity is an important public health problem that contributes to excess morbidity and mortality world-wide [1,2]. Obesity (30< BMI), and overweight (25 < BMI < 30) are major risk factors for morbidity and mortality. Calculations based on disease and gender specific relative risks from a recent meta-analysis of internationally published data [3] adjusted to Israeli demographic data, estimated that obesity and overweight and account for around 3,105 deaths (95%CI 2,415-4,266) per year in Israel (7.7% of all deaths). After adapting Canadian Cost of Illness [4] data to Israeli economic conditions, the costs of treating morbidity due to obesity and overweight in Israel amount to NIS 1.92 billion (95%CI: 4.29-8.33), equal to 0.24% (95% CI 0.18%-0.35%) of the GDP, or 3.05% (95% CI 2.29%-4.45%) of Israel's health expenditures. Additional costs of NIS 1.89 billion have been estimated to be due to productivity losses, and a further NIS 1.95 billion results from other indirect costs.. Thus the total costs attributable to obesity and overweight amount to approximately NIS 5.76 (95%CI: 4.29-8.33), equal to about 0.73% (95%CI 0.54%-1.05%) of the Israeli GDP.

In 2005, the Ministry of Health established the Healthy Israel 2020 initiative to develop a national preventive health targeting program for Israel. Twenty committees covering a broad spectrum of health domains were formed. The Health Behaviors Committee created eight subcommittees, each dealing with key lifestyle behavior risks such as obesity, tobacco use and sedentariness, among others. Health burden-based objectives, quantifiable targets, and science-based and practicable interventions were selected to reduce the respective health (and economic) burdens. A National Prevention and Health Promotion Program (NPHPP) to control obesity, encourage healthful nutrition, and enhance physical activity has been jointly developed by the Ministries of Health, Education, and Culture & Sports.

The NPHPP interventions to prevent and control obesity can be broadly grouped into three domains:

A. The clinical sphere. Examples include screening, counseling, and medical interventions [such as the prescription of Orlistat, the only currently certified weight loss medication], as well as surgical interventions

B. The community. Examples include home, school, workplace, restaurant and supermarket- based interventions, media strategies, and other initiatives initiated by community-based organizations such as municipal governments and NGO's

C. The legislative sphere. Examples include taxation of unhealthful food products [5], provision of government subsidies to reduce the price of healthful foods [and vice-versa] [6], mandatory food labeling [7], and prohibiting the sale of unhealthful foods in vending machines [8,9].

Nationwide community or clinic-based interventions to reduce obesity can be costly due to the large numbers of people targeted. These high intervention costs form a barrier to the adoption of such programs by government funding agencies (e.g., Ministries of Finance). However, adoption of such programs have the potential not only to generate additional QALYs (quality adjusted life years) but to also reduce treatment costs for diseases directly associated with obesity (e.g., diabetes, hypertension, etc.). In addition, it is likely that further savings will accrue due to productivity gains secondary to decreased presenteeism and absenteeism. Further monetary savings should accrue on account of reduced costs of informal care givers in the community, delayed burial costs, and other indirect costs.

While adding extra QALYs is of paramount importance, interventions that are cost-saving (viz., averted direct and indirect costs exceed intervention costs) or whose net cost (i.e., intervention cost less direct and indirect savings) is low are the ones more likely to be funded.

Recent publications [10-12] have prioritized these interventions by calculating their cost-effectiveness ratios in the form of costs per QALY.

On the other hand, cost-effectiveness analyses of fiscal incentives such as subsidizing healthful foods or taxing unhealthful ones and legislative interventions such as food labeling or banning the sale of unhealthful products in school vending machines were not found in the international scientific literature.

This study then seeks to calculate the expected costs and cost-utility of the implementation of various clinical and community-based intervention strategies forming part of the interventional suite currently recommended by the tri-ministerial NPHPP to reduce the burden of obesity in Israel.

## Methods

### Calculation of excess health costs by BMI category

Sixteen studies from various countries and time periods that reported on the relationship between Body Mass Index (BMI) and health service expenditures [13-28] were identified. The reported data were interpolated and indexed to a standard scale where an average normal BMI value of 22.5 was given an index value of 100. The average of each BMI specific value was then taken to provide a combined index relating health service expenditures with BMI levels.

The BMI prevalence distribution for adults aged 20 and above [Personal Communication, Dr Tami Shohat, Israel Centre for Disease Control] was multiplied by the total adult population aged 20 and above in 2010 of 4,835,024 persons [29,30] in order to estimate the number of adults within each unit BMI category.

The estimated total of 1.92 billion NIS additional health care costs attributable to obesity (1,145 million NIS) and overweight (773 million NIS) were distributed over the entire BMI spectrum in proportion to the number of persons and the excess health care costs in each BMI unit category. Both total costs and average costs per person in each BMI category were calculated, enabling us to calculate the marginal change in health care costs per unit change in BMI. All costs are presented in New Israeli Shekels (NIS) at mid-2010 price levels (Exchange rate 3.729 NIS to the US dollar).

### Allocation of persons to cost-saving and very cost-effective interventions

All eight office-based and community and dietary interventions [10,31-34] that had been identified as being either cost-saving or very cost-effective in a previous comprehensive study of the cost-utility of interventions [10] were selected in order to estimate their impact on costs when implemented on a national level as part of the NPHPP. Brief descriptions of the interventions are provided in Appendix 1. Two studies that had been identified as being very cost-effective were not included among the eight selected studies on the grounds that one [35] was based on the combined results of a prior included study [32] and the other was based in a clinical drug trial setting [36] that cannot be replicated in routine public health interventions.

It was assumed that the 52,517 severely obese (BMI ≥ 40) persons would be allocated equally between the standard and low carbohydrate dietary interventions [34]. The remaining 4,782,506 persons in the population would be distributed equally (i.e., 797,084 persons per intervention) among each of the other six interventions. Therefore we are creating an estimate based on the assumption that Israel adopts a multiplicity of interventions (that have previously succeeded abroad) in equal numbers.

In the intervention where initial screening costs for identifying persons who were overweight or obese were omitted [33], a cost of 5.4 NIS per person screened was added, based on the estimated Israeli costs of three minutes screening time (50% by physicians and 50% by nurses) [10] Full details of the intervention specific cost and QALY estimates are given in our companion source paper [10].

### Costs per QALY definitions

The costs of the interventions were adjusted to Israeli NIS by the country-specific consumer price index to 2010 price levels and by the appropriate exchange rate which took into account purchasing power parities (PPP) for the non-tradable [e.g., manpower] elements of the interventions. Costs per QALY gained were calculated by indexing the results of a prior comprehensive cost-utility study based on 2008 price levels [10] to 2010 price levels [30]. An intervention was defined as being cost-saving if it yields actual savings as well as contributing additional QALYs, very cost-effective if the cost per QALY is less than the Israeli per capita GNP of NIS 104,161 in 2010 [37] or cost-effective [if the cost per QALY is between 1-3 times the per capita GNP (NIS 104,161-312,483). If the cost per QALY is more than three times the GNP per capita (> NIS 312,483) then the intervention is regarded as not being cost-effective.

### Calculation of treatment cost savings by BMI level

Total (gross) intervention costs were calculated by multiplying the numbers targeted by the unit costs of the intervention, thereby implicitly assuming an ideal coverage rate of 100%. Absolute effects of the interventions on BMI were calculated directly from the specific study reports on changes in BMI [18,32,33], or indirectly via weight changes [35] and QALYS lost from morbidity due to elevated BMI [10]. Lifetime savings in health treatment costs by intervention were based on the marginal health care costs saved per BMI change multiplied by the intervention-specific BMI change. Future years' savings in health expenditures were calculated using a 3% per annum discount rate.

There is a paucity of data on long-term recidivism rates after the cessation of intervention programs against obesity. The efficacy data in the studies that were used in this paper were based on intervention studies of up to only one year duration, with modeling techniques in some studies extrapolating the outcomes over longer periods of time.

A baseline 50% per annum recidivism rate, was selected (with a sensitivity analysis performed in the range of 20%-80% per annum) as a conservative estimate based on the following information:

i) An implied recidivism rate of 4.0% per annum was derived from a meta-analysis of studies using diet alone, which reported an average weight loss of 4.5 kg at six months, which was reduced by only 13% to 3.9 kg after four years [38].

ii) Studies using diet and exercise reported a 7.9 kg loss at six months that was reducedby 33% to 4.5 kg after four years- implying a 15.0% recidivism rate [38].

iii) The successful maintenance of a weight loss of 7% or more 2.8 years after an intensive lifestyle intervention by 37% of overweight individuals with impaired glucose intolerance [39] - implying a recidivism rate of 30.2%.

iv) A reported 13%-20% of persons who maintained a weight loss of at least 5 kg for five years [40]. This is equivalent to an annual recidivism rate between 33%-40%.

v) Data from the US national weight control registry indicate a one-year recidivism rate of 35% (defined as gaining 2.3 kg or more), while 59% maintained their body weight and 6% continued to loss more weight [41].

vi) Research indicating that approximately 20% of overweight individuals are successful at long-term weight loss, defined as losing at least 10% of initial body weight and keeping the weight off for at least one year - implying an 80% recidivism rate [40,42].

### Indirect costs

Averted productivity losses were based on 98.8% of estimated direct health care savings [4]. Similarly, averted care costs and other indirect costs were estimated to be 80% and 22% of productivity losses [4]. Net costs to society were calculated by deducting all these averted direct and indirect costs from the gross intervention cost. A sensitivity analysis was performed on a range of recidivism rates from 10%-90% per annum.

### Converting BMI changes to QALY gains

BMI unit values were assigned QALY weights by interpolating the values (Table 1) from a UK study [34]. This allowed for marginal QALY improvements per unit decrease in BMI and hence total lifetime QALY gains (discounted at 3% per annum) and cost per QALY per intervention to be calculated. Additional life expectancy per BMI change (0.415 years per unit BMI) was based on the average value extracted from two studies [33,35] that reported very similar values of an additional 0.41 and 0.42 extra life years per unit BMI decrease, based on reported increased life expectancy and BMI decreases [30] and QALY increases [35]. These intervention specific estimates of increased life expectancy were then converted to QALYs by adjusting by the appropriate Health Adjusted Life Expectancy (HALE) multipliers (that take into account the background decrease in functioning due to aging) and the appropriate BMI to QALY ratio [43].

**Table 1:** QALY Weights by BMI Level

BMI (kg/m^2^)	Description	QALY Weight
20 or less	Underweight	0.741
20 to 24.9	Desirable	0.787
25 to 29.9	Overweight	0.769
30 to 34.9	Obese Class I	0.707
35 to 39.9|	Obese Class II	0.672
≥ 40	Obese Class III (morbidly/extremely obese)	0.624

On an assumption (based on expert opinion as to the ideal age group for intervene with weight control programs) that the average age of a participant in the program was 52 years, and that each had, on average, a residual life expectancy of 30 years [30], we estimated the additional (discounted) health-care costs due to the increased life expectancy of those persons who reduced their BMI.

Excess years gained due to the interventions were assumed to have the characteristic of having average health costs of the treated targeted cohort (i.e., overweight/obese or severely - obese).

## Results

Overweight individuals bore health care costs that were 12.2% above the those of persons with normal or below-normal weight to height ratios (BMI < 25). This percentage rose to 31.4% and 73.0% for obese (30 ≤ BMI < 40) and severely obese persons (BMI ≥ 40), respectively (Table 2). For example, an obese person with a BMI of 37.5 would incur an additional 1,672 NIS in health care expenditures per year. If, however, as a result of participation in an intervention program the person's BMI drops by 1 kg/m^2 ^to 36.5, their health expenditures would be expected to fall by 109 NIS (i.e.,: 1,672-1,563) per year (Table 1).

**Table 2:** Health Care Costs (NIS at 2010 price levels) by BMI

BMI	Health Care Costs	BMI distribution	Population	Health Care Costs of Obesity or Overweight	
				Total	per person
	(a)	(b)	(c)	(d)	(e)
< 19	n.a.	2.4%	115,576	n.a.	n.a.
19	94%	2.5%	121,863	0	0
20	96%	4.6%	220,030	0	0
21	98%	5.3%	258,233	0	0
22	100%	7.2%	345,761	0	0
23	103%	8.7%	420,717	0	0
24	106%	9.8%	473,911	0	0
25	108%	9.8%	473,427	123,593,389	261
26	110%	8.9%	429,905	145,645,761	339
27	112%	8.2%	395,570	156,151,524	395
28	115%	7.2%	346,729	171,456,620	494
29	119%	6.0%	292,084	175,690,848	602
30	123%	4.9%	238,890	196,332,658	822
31	126%	3.1%	149,911	141,832,838	946
32	129%	2.4%	116,060	123,015,268	1060
33	133%	2.5%	120,412	144,311,733	1198
34	137%	1.4%	66,734	88,051,773	1319
35	140%	1.5%	72,054	103,825,223	1441
36	143%	1.1%	51,117	79,881,318	1563
37	146%	0.78%	37,813	63,208,344	1672
38	149%	0.39%	18,906	33,668,568	1781
39	154%	0.35%	16,806	32,499,750	1934
40	158%	0.23%	11,204	23,329,496	2082
41	162%	0.12%	5,602	12,496,246	2231
42	166%	0.19%	9,103	21,657,584	2379
≥ 43	184%	0.55%	26,609	80,664,301	3031
			
		100%	4,835,024	1,917,313,242	
			
	n.a.= not available				

Notes:

(a) Based on the average of sixteen articles [13-28].

(b) Calculations based on Israeli survey data (Personal communication. Dr T. Shohat).

(c) Column (b) multiplied by 4,835,024 persons [29,30] aged over 20

(d) Excess costs ((a) less 100%) multiplied by population (c) and factors that result in a total of 772,538,141 NIS and 1,144,775,101 NIS [4] additional health care costs attributable to overweight and obesity, respectively.

(e) Column (d)/Column (c)

For overweight individuals and obese persons with BMI < 40, costs per person for the interventions (including the screening overhead) ranged from 35 NIS (for a multifaceted community intervention) to 860 NIS (Table 3), for an intensive clinical setting intervention, including the unit costs of the professionals carrying out the intervention (e.g., dietician). The interventions ranged from being cost-saving to being a very cost-effective 50,543 NIS per QALY. Expected average BMI decreases ranged from 0.05 to 0.90 (Table 3). Higher intervention costs, higher costs per QALY, and larger BMI decreases characterized the two clinical lifestyle interventions for the severely obese (BMI ≥ 40) (Table 2).

**Table 3:** Costs (NIS at 2010 price levels) and Effects of Dietary Interventions to Reduce Obesity.

Intervention (for fuller description See Appendix 1)	Study	Ref		Cost per Person	Cost per QALY	BMI decrease
25 ≤ BMI < 40					(f)	
*Clinical setting*						
*Nutritional counseling *						
Various staff providing nutritional counseling over one year, modeled over lifetime	Ginsberg	[10]	(a, d)	57	50,543	0.05
General practitioner-based nutritional counseling over one year, modeled over lifetime	Olsen	[33]	(c)	395	4,379	0.22
Dietician-based nutritional counseling, over one year, modeled over lifetime	Olsen	[33]	(c)	860	31,982	0.07
Clinic-based, intensive lifestyle counseling for the obese over 3 years modeled over lifetime	J-van der Bruggen	[32]	(c, e)	798	26,769	0.90
*Combined nutritional counseling and office support *						
Counseling by primary care internists trained in nutrition counseling with an office-support program tracked for one year Cost-utility estimates based on Israeli data	Ockene	[31]	(b, d)	74	< 0	0.81
***Community setting***						
Community-based strategy using mass media campaigns, social support, risk factor screening and counseling in various settings over 5 years modeled over lifetime	J-van der Bruggen	[32]	(c, e)	35	19,938	0.15
**BMI > 40**						
*Clinical setting*						
Medical center staff counseling to adhere to a low carbohydrate diet over one year. Time horizon for cost utility analysis also one year	Tsai 2003	[34]	(c)	5,767	39,591	1.59
Medical center staff counseling to adhere to Standard NCEP 1 diet [g] over one year. Time horizon for cost utility analysis also one year	Tsai 2003	[34]	(c)	5,584	38,759	0.97

Notes: CS = Cost Saving

(a) Based on primary Israeli epidemiological, demographic and cost data.

Effectiveness from meta-analysis of international studies.

(b) Based on primary Israeli epidemiological and demographic data.

Cost data updated and converted to Israel 2010 price levels. Effectiveness from international study.

(c) Based on international data converted to Israel 2010 price levels.

(d) Based on cost per QALY at average 10 year AMI risk of 12.8% at age 55.

(e) Includes initial screening charge of 5.4 NIS per head [10].

(f) Ref [10] updated to 2010 price levels. All values are ACERs, average cost-effectiveness ratios.

Implementation of the NPHPP to cover the entire Israeli population aged over 20 years with a combination of six different interventions would cost 2.07 billion NIS (Table 4). Approximately 201,000,000 NIS would be recouped in the form of decreased treatment costs and a further 199,000,000 NIS would be regained via increased productivity and decreased absenteeism. Other averted indirect costs account for the saving of an extra 202,000,000 NIS. The discounted health costs attributable to additional life expectancy as a result of BMI reductions, amount to approximately 89,000,000 NIS. Therefore, the total net costs of the interventions to society will be 1.55 billion NIS.

**Table 4:** Intervention Costs and Savings (at 2010 price levels).

	Ref	Numbers Targeted	Total Intervention Costs	Lifetime Savings in Direct Costs of Illness	Lifetime Savings in Productivity costs	Lifetime Savings in Carer Costs	Lifetime Savings in other Indirect Costs	Medical Costs due to increased longevity	Net Costs
Ginsberg	10	797,084	45,119,420	4,648,779	4,592,029	3,656,496	1,016,092	2,013,874	33,219,898
Ockene	31	797,084	58,750,777	71,154,890	70,286,270	55,966,868	15,552,459	30,824,650	-123,385,060
J-van der Bruggen I	32	797,084	27,975,847	13,176,831	13,015,976	10,364,235	2,880,085	5,708,269	-5,753,012
J-van der Bruggen II	32	797,084	635,754,990	79,060,989	78,095,855	62,185,409	17,280,510	34,249,611	433,381,838
Olsen I	33	797,084	315,120,819	19,714,059	19,473,401	15,506,090	4,308,939	8,540,228	264,658,558
Olsen II	33	797,084	685,335,609	5,877,750	5,805,998	4,623,143	1,284,711	2,546,270	670,290,278
Tsai I	34	26,259	151,434,528	4,611,477	4,555,182	3,627,156	1,007,939	3,427,595	141,060,368
Tsai II	34	26,259	146,623,901	2,803,055	2,768,836	2,204,742	612,669	2,083,440	140,318,039
		
TOTAL		4,835,024	2,066,115,891	201,047,829	198,593,546	158,134,140	43,943,405	89,393,937	1,553,790,908

Baseline case of 50% recidivism per year.

If the recidivism rate is reduced to 35% or 20%, net societal costs will fall to 1.35 billion and 0.89 billion, respectively. On the other hand, if recidivism rates reach 65% or 80%, net societal costs will increase slightly to 1.67 and 1.74 billion NIS, respectively (Table 5).

**Table 5:** Net Costs per QALY of Interventions by Recidivism Rate.

Recidivism Rate	20%	35%	50%	65%	80%
Intervention Costs	2,066,115,891	2,066,115,891	2,066,115,891	2,066,115,891	2,066,115,891
***less Savings ***					
Direct Treatment	-462,448,210	-280,406,001	-201,047,829	-156,699,045	-128,379,941
Productivity Losses	-456,802,895	-276,982,958	-198,593,546	-154,786,149	-126,812,748
Carer Costs	-363,738,572	-220,553,299	-158,134,140	-123,251,611	-100,977,223
Other Costs	-101,078,183	-61,288,872	-43,943,405	-34,250,008	-28,060,247
***plus longevity Costs***	205,623,043	124,679,767	89,393,937	69,674,687	57,082,876
	
Total Savings	-1,178,444,816	-714,551,362	-512,324,983	-399,312,125	-327,147,283
	
Net Costs	887,671,075	1,351,564,529	1,553,790,908	1,666,803,766	1,738,968,608
	
QALYS saved	75,150	45,567	32,671	25,464	20,862
	
Net Costs per QALY	11,812	29,661	47,559	65,457	83,355

In the baseline 50% recidivism scenario, 32,671 QALYs would be saved by implementing the interventions, at an average cost of 47,559 NIS per QALY (Table 5), less than half the per capita annual GNP [24]. Decreased recidivism rates of 35% and 20% will increase the numbers of QALYS saved to 45,567 and 75,150 respectively, and decrease costs per QALY to 29,661 and 11,812 NIS per QALY respectively.

Increased recidivism rates of 65% and 80% will decrease the numbers of QALYS saved to 25,464 and 20,862 respectively, while increasing costs per QALY to 65,457 and 83,355 NIS per QALY (Table 5), which are both considered as being very cost-effective ratios.

## Discussion

In the baseline case, assuming an annual recidivism rate of 50%, the total intervention costs of eight weight loss programs aimed at the entire adult population of Israel, amount to 2.07 billion NIS. The costs of such a large program are likely to be spread over a period of time, say five or ten years, due to manpower and budgetary constraints. Approximately 29.1% of these costs would be offset by averted direct and indirect costs attributable to obesity. However, after treatment costs due to increased longevity are taken into account, these saving would be reduced by only about one-quarter (24.8%) of the intervention costs.

Our assumption of 100% coverage is a reasonable one given Israels small size and universal health insurance coverage. In reality, compliancy with some programs will fall short of 100%, but this has been implicitly recognized in the cost and QALY values taken from the source studies (10).

Our assumption of a continual geometric decrease in recidivism could also be considered as being conservative since weight loss maintenance may get easier over time; after successfully maintaining weight loss for 2-5 years, the chance of long-term success generally increases [40,41].

A sensitivity analysis on recidivism rates showed the total net cost is relatively inelastic with respect to changes in the recidivism rate above the baseline 50% levels; a 60% increase in recidivism (increasing the rate from 50% to 80%) only causes a 12% increase in the net costs to society. However, reducing recidivism by 60%, (reducing the estimate from 50% to 20%), which may be achievable by adding a maintenance component to existing programs, would have a more elastic impact on costs, reducing them by 43%. Sixty percent increases and decreases n recidivism would reduce and increase QALY gains by 44% and 65%, respectively.

A growing body of literature supports the notion that the factors leading to recidivism may be different and more challenging than those faced during the initial months to one year of weight loss [44]. Therefore the prevention of recidivism merits separate intervention studies (such as maintenance therapy) and cost-effectiveness analyses.

The effectiveness (and resulting cost-effectiveness ratios) generated by the interventions listed in this paper may be overstated or understated. The seven non-Israeli programs were modeled using observations and assumptions germane to the health care systems and cultures in which they were performed. Hence they may not be completely relevant to the Israeli setting. This may even be true to some degree with respect to the lone Israeli-based screening and dietary intervention [10], as it predicated upon a meta-analysis of the effect of interventions on LDL cholesterol in international studies.

On account of the high intervention costs but relatively low cost savings, readers of this study could conceivably decide not to field the proposed suite of interventions. Yet further examination reveals that although only one of the eight interventions is cost-saving [31] the others are all very-cost effective (even when assuming high recidivism rates) and are therefore worth adopting. They compare very favorably with the most cost-effective interventions identified to reduce damage from smoking [11] and to enhance physical activity [12]. In addition, the gain of roughly 32,671 QALYs, which reflects decreases in morbidity and mortality, should be considered a highly desirable benefit of the proposed intervention program.

A recent OECD document confirmed that counseling people at risk in the primary care setting was one of the most effective and cost-effective ways of improving health [45]. If a long-term time perspective is used, other interventions such as school-based interventions, food advertising regulation, worksite interventions, food labeling and mass media campaigns may also be considered cost-effective with respect to their impact on obesity. In addition, fiscal measures, such as taxing unhealthful foods would be a cost-saving intervention that increases quality of life without incurring extra costs to society [5]

Finally it should be emphasized that current international and HI2020/NPHPP recommended interventions for weight loss include both dietary and physical activity (PA) components. A companion study will be addressing the latter, and it is expected that the insights of both studies will be merged to provide comprehensive CUA data for the complete suite of interventions.

## Conclusions

Fielding an eight-pronged combined clinical and community-based dietary interventional program within the context of the National Program for Health Promotion and Prevention would save over thirty-thousand quality-adjusted life-years, and is very cost-effective. Yet implementation would require a substantial long-term monetary investment by the government. The approximations and extrapolations conducted on the basis of non-Israeli data support future studies geared towards the generation of Israeli epidemiologic and cost modeling data. That said, the overall very cost effective cost-utility estimate of this analysis strongly supports the adoption of the proposed national program to combat obesity.

## Appendix 1: Dietary interventions

### Model incorporating Israeli obesity control data

Ginsberg [10] constructed an intervention model estimating the cost per QALY of dietary interventions [using reductions in LDL-C levels as an intermediate marker] on the basis of primary Israeli data. It was assumed that everyone aged 20 and above [n = 4,633,593] would have their waist circumference and BMI measured. The 14.8% [685,772 obese persons] with a BMI ≥ 30 would then receive dietary counseling. The cost of the dietary intervention was based on the client making two visits during the first three months and then monthly visits thereafter. Based on common Israeli practice, it was assumed that 50% of the visits would be to a physician, 25% to a trained nurse, 20% to a dietician, and the remaining 5% to a psychologist.

### International interventions adapted to the Israeli setting

In the USA, Ockene [31] evaluated the effect of nutritional counseling offered by primary care internists offered training in nutritional counseling and aided by an office-support program on the saturated fat intake, weight, and serum lipid measurements of hyperlipidemic patients over one year. Net costs per reduction in LDL-C levels were calculated. The model developed for the Israeli dietary intervention [4], was then used to calculate the resultant costs per QALY of the intervention.

Van-der Bruggen's Dutch lifestyle intervention model [32] projected the costs and effects of both a community-based lifestyle program for the general population and an intensive lifestyle intervention for obese adults, implemented in a health care setting.

Olsen's Danish study [33] randomized 60 GPs either to provide nutritional counseling or to refer patients to a dietician for counseling.

### Severely Obese

Tsai et al. compared a low-carbohydrate diet (under 30 grams/day) with the National Cholesterol Education Program Step I low fat diet for treating severe obesity [34] in a medical center setting. Study participants received weekly group counseling sessions during the first month, followed by monthly group sessions for the next five months.

## Competing interests

Both authors are salaried staff of the Ministry of Health and there are no competing interests to declare.

## Authors' contributions

GMG designed the study, collected the data, carried out the data analysis, wrote the initial and wrote, read and approved the final manuscript. ER contributed to the interpretation of the data, made critical revisions and wrote, read and approved the final manuscript.

## Authors' information

Gary Ginsberg is a health economist, specializing in epidemiologic-economic evaluations (cost-utility analysis), and is the Director of the Medical Technology Assessment Sector of the Israeli Ministry of Health. He previously worked for the WHO, RTI and the London Borough of Wandsworth.

Elliot Rosenberg is a physician specializing in General Preventive Medicine and Public Health. His interests lie in national health targeting, clinical preventive medicine, health promotion in the worksite and in elders, and alertness management. He serves as National Coordinator for the Healthy Israel 2020 initiative and is Head of the Department of Occupational Health at the Israeli Ministry of Health and is a member of the Israeli Task Force on Clinical Preventive Services. He served for many years as a career flight surgeon in the Israeli Air Force.
